# Government sectoral spending and human development in Nigeria: Is there a link?

**DOI:** 10.1016/j.heliyon.2023.e17545

**Published:** 2023-06-26

**Authors:** Ademola Andrew Onabote, Bright Onoriode Ohwofasa, Rotimi Ayoade Ogunjumo

**Affiliations:** a Department of Economics, Landmark University, Omu-Aran, Nigeria; b Department of Social Sciences, Delta State Polytechnic, Ogwashi-Ukwu, Nigeria

**Keywords:** Government sectoral spending, ARDL model, Nigeria

## Abstract

The continuous increase in government expenditure in the last three decades without a commensurate improvement in all known indicators of development has generated heated debates among scholars as to the justification for the persistent rise in the annual expenditure of the government. Therefore, this study examined the effects of government sectoral spending on human development in Nigeria using annual data spanning the period 1986–2021. This study contributed to the literature by examining the effects of government sectoral spending on human development using a robust human development index that captures the multifaceted state of economic development in terms of educational attainment, life expectancy and per capita income, unlike previous studies that concentrated on aggregate government spending and used the gross domestic product as an indicator of development. Surprisingly, however, results from the Autoregressive Distributed Lag (ARDL) model employed indicated that both in the short and long run, there is no link between government sectoral spending and human development in Nigeria. Although, outcomes from ECMs suggest that government sectoral spending may affect human development in the long run.

## 1 Introduction

In almost all stages of development, government expenditure plays a crucial role that enables the economy satisfactorily function irrespective of the type of economy whether less-developed or developed nations. The current emphasis being placed on using government expenditure as a vehicle to fast-track economic growth and development is occasioned by the market failure of the 1920s that was earlier advocated by classical economists.

Contrary to the classical belief, there were falling demands predicated on over-production with the result that unemployment was inevitable leading to a decline in output and income [1]. The less-than-full employment that occurred made the laissez-faire policy of classical economics be questioned and the subsequent emergence of Keynesian economics advocated for a central body such as the government to spend to stimulate the economy. The Great Depression of the 1930s as well as the emergence of Keynesian writers on the world scene drew the attention of most nations' governments to the efficacy of using government spending to regulate and stabilise the aggregate general economy [2]. Accordingly, over the last three decades, government spending in most nations of the world has increased significantly. However, while there is a significant improvement in global economic growth and development, several sub-Saharan African countries particularly Nigeria continue to experience rising levels of poverty and lower level of development outcomes which culminated in the World Bank's classification of Nigeria as the global capital of extreme poverty [3]. Although the total government spending in Nigeria increased extensively in the past decades, for instance, it increased from N16.2 billion in 1986 to N4988.8 billion in 2014 and in recent times, increased tremendously due to the recent COVID-19 pandemic but sadly, the country is far from being developed and her economic growth has remained sluggish. For instance, it averaged about 2.0% between 1986 and 2021 [4]. In this manner, the relationship between government spending and economic development has remained a dominant and persistent theme in the country particularly, among academics and in policy circles.

Essentially, human development is concerned with an increase in the level of welfare of human and most importantly the environment that enable human life to have meaning thereby reflecting the level of dignity [5]. As human constitutes a key factor in production processes, natural resources will be meaningless if there were no humans that will work on them so that inputs are converted into output.

The concept of the human development index (HDI) developed by the United Nations as an appropriate measure of socioeconomic well-being has come to be regarded as an alternative to the traditional gross national product or gross domestic product. The HDI which is built upon Sen's capabilities approach is predicated on the belief that the traditional one-dimensional measure of socioeconomic well-being is unreliable and misleading [6]. In his submission [7], defined the HDI as “a composite index that measures the average socioeconomic achievements in a country in terms of three core capabilities: the capacity to realize a long and healthy life; access to knowledge; and a decent standard of living”.

The question about how to achieve a sustainable level of development that will encompass all of the core features of the human development index does not merely requires growth, but growth that reflects distributional changes which in turn translate to unemployment and poverty reduction, increase in school enrolments and high literacy rate leading to higher welfare. This is the genesis of the debate sparked in the development literature concerning the factors that affect the human development index [[8], [9], [10]]. With regard to the debate, the direction and nature of government spending have been fingered as one key factor. Meanwhile, the empirical evidence supporting this view has been scarcely pursued. Some country-specific and cross-country studies have been conducted to unravel the relationship between the human development index and government spending. Notably, a number of these studies are sector specific (see for example [[9], [10], [11]]). Accordingly, the results of these studies on the effect of government spending on human development have been mixed: significantly positive or negative and statistically insignificant.

Thus, the role of government spending in the quest for sustainable development is still open to debateAs a result, the existing literature is replete with studies of government spending and economic growth (see, for example [[12], [13], [14], [15], [16]]), and the current study is motivated by replacing the traditional GDP with a human development index developed by the United Nations. Our belief in using HDI, which is based on Sen's capabilities approach, stems from our conviction that the traditional one-dimensional measure of socioeconomic well-being is unreliable and misleading [6,7]. Furthermore, our motivation is fueled by the fact that the majority of studies on the relationship between government spending and economic growth are sector-specific [[18], [19], [20], [21]]. The current study departs from the aforementioned in that it is disaggregated into a multi-sector approach such as government spending on administrative services, economic services, social and community services as well as transfers. Accordingly, the effects of the various sectoral spending on HDI are considered using a relatively recent econometric technique of autoregressive distributed lag (ARDL) model developed by Ref. [22]. An approach whose empirical probe has been scarcely pursued in the extant literature.

Although, some empirical studies have analyzed the effect of government spending on economic development findings are mixed. There were authors whose findings showed that government spending exerted a positive impact on development [13,23,24]. There were others whose findings revealed a negative association between the variables [2]. Still, some other studies could not establish any relationship [1,25].

Nevertheless, these past studies are characterized by a major shortcoming. These studies focused on government spending and the gross domestic product (GDP) nexus. However, evidence from Ref. [26] suggests that gross domestic product alone as an indicator does not address the fundamentals of living standards. Besides, existing studies also worked on the assumption that the response of economic development to changes in total government spending is the same. Sadly, this makes it impracticable to assess how government sectoral spending has independently affected economic development.

In our bid to fill this lacuna, the study is posed to scrutinise the relationship that exists between government sectoral spending and human development in Nigeria using the human development index (HDI) data covering 1986–2021. The HDI takes into account the complex multidimensional state of economic development in terms of educational attainment, life expectancy and per capita income. The findings from our study showed that both in the short and long run, there is no link between government sectoral spending and human development in Nigeria. This article is divided into four parts. Section one covers the introduction, and the presentation of related literature is done in section two. The methodology is unveiled in section three while section four presented the discussion of empirical results. Finally, the paper is concluded with policy implications based on the findings in section five.

## 2 The literature

### 2.1 Theoretical issues

The theoretical literature on the relationship between government spending and economic growth abounds [27]. suggested the ratio of GNP in the public sector as a means of increasing state ability. Although [27], was not initially presented in the form of a theory as it was not particularly clear if Wagner was comparing the growth of public expenditure to GNP. However [28], gave the interpretation of Wagner's theory to mean the relative size of the public sector growth. Therefore, the theory is being conceptualized to mean that the growth in per capita income in an economy will expectedly lead to an increase in the relative size of the public sector.

[29] accepted Wagner's theory but rejected his conclusion on the continuous expansion of the public sector occasioned by an increase in GNP per capita. Their argument regarding public spending was based upon a political postulation that the wish of government is to always spend more whereas the citizens have the habit of shinning away from paying more taxes [29]. submitted that the ideal thing is for government to listen to the yearnings of the people to find a solution to the spending deficit.

Nevertheless, according to Ref. [30], the growth of an economy is due to the interactions of several elements in the production process. These elements are fundamentally labour and capital which interact with technology to produce output. Using this model, neoclassical economics opined that the only condition to maintain a positive growth rate is to ensure that the growth of the population is such that it can offset any decreasing return to capital accumulation. Alternatively, changes in technological progress could cause constant upward shifts in the marginal productivity of capital. In a balanced growth equilibrium, the stock of capital is not expected to depreciate while the growth rate of the population should not exceed that of capital and output.

In the case of human development, the assumption behind the theory surrounding the HDI is that individuals would prefer the maximization of any achievements that will reflect the three core capabilities measurement that entail the functioning which the said capabilities allow for [7,8]. The core of the human development index which is measured by life expectancy at birth, adult literacy and enrolment into primary, secondary and tertiary education levels is what Sen referred to as capabilities. For Sen, a weight of one-third is attached to each of the three achieved capabilities and each country's GDP is converted into a common currency to align with purchasing power of the country's GDP. The implication of an individual country's GDP purchasing power parity is that a higher HDI implies a higher well-being of the citizens. Sen attached heavy weight to the per capita GDP to stress the critical role played by material welfare either as an essential ingredient of a good life or the necessary capability needed to realize additional requirements of a good life. The underlining theoretical underpinning of the capabilities hypothesis is that a higher capabilities level is sine-qua-non with achieving relatively higher levels of material welfare [7,17].

## 2.2 Empirical issues: a brief review

A number of studies were conducted to ascertain the relationship between human development index and government spending in health and education and mixed findings were documented. These authors include [5], [31], [32], [33], [34], [35]. These studies mainly focused on education and the health sectors and therefore are limited in scope. An array of other studies considered other sectors of the economy in their analysis of government spending and growth and or human development index.

Thus [36], modelled human development as a function of government capital expenditure, government recurrent expenditure on health, education, agriculture, potable water resources, rural development, housing, environmental protection and energy. Employing the technique of ordinary least square (OLS) covering the period 1999–2012, the study found among other things evidence of a significant positive effect of government spending on human development in Nigeria. In a study conducted in India [9], employed the ARDL model on data covering 1990–2018 to assess how government spending on health and education can affect human development. The study found that human development is significant and positively responsive to changes in government spending on health contrarily to its significant negative changes in spending on education in the long run. However, the study found different outcomes in the short run in that no relationship could be established between human development and the explanatory variables who were statistically insignificant. These findings prompted the study to suggest an increase in funding for education and health sectors by the Indian government.

[10] averred that the world economy in recent times has been saddled with volatilities amidst calls for the increased injection of funds into the health sector by governments of Economic Community of West African States (ECOWAS) members. The study which employed the panel ARDL technique found that per capita health expenditure is determined by population growth and economic uncertainty in the long run. The study further found that as the model was segregated into different income groups by the participating countries, it was observed that a negative relationship between spending on health and economic uncertainty was evidenced in low-income economies in the short run as well as the long run where population exerted a negative impact on per capita spending on health in lower-middle-income economies within the ECOWAS region. The study concluded that the reliance on the government in funding the health sector appears unsustainable and therefore calls for the need of funding the sector through private and public sector initiatives.

However, the findings of [9,10] were inconsistent with the outcomes of the study conducted by Ref. [11] who focused on the extent to which development spending affects gross domestic product and human development index in seven least-income states which include Uttar Rajasthan, Jharkhand and Pradesh among others in India. The study concluded that although development spending may not have exerted a significant effect on HDI, improvement in health facilities is near impossible without increased spending in the health sector. Comparatively, this study is relevant to Nigeria's situation where government spends more in rural areas without noticeable outcomes and where the health sector in Nigeria is constantly beginning for attention.

In a recent study conducted by Ref. [37] in Indonesia, he examined how a shift in public expenditure occasioned by the Covid-19 pandemic affected changes in the decentralization of funds in East Java province of Indonesia. A cross-section of data from 38 districts was modelled using linear multiple regression techniques. Essentially, the study could not find any noticeable change in the method of fund decentralization, human development index as well as civil capital expenditure on civil service. However, the study noted that vertical fiscal imbalance had a significant positive effect on economic growth before the Covid-19 pandemic, and no effect during and after the deadly disease period was over. The study, however, did not carry out an assessment of the direct relationship between sectoral spending and the human development index.

In their paper [38], used data from low-income economies covering 25 SSA countries to evaluate the degree of response in economic growth to changes in government spending. Accordingly, findings indicated an enhancement in the economic growth of SSA low-income economies occasioned by a rise in public expenditure. However, when efficiency interacted with the component of government spending, the study could not find evidence of a significant response in growth to changes in government spending [39]. assessed the causal influence of government spending on economic growth using an array of samples comprising less-developed and developed economies. Accordingly, it was observed by the study that causality runs from growth to public expenditure. In addition, a bidirectional causality running from public spending to growth and vice versa was also reported.

In a recent study [2], scrutinized the effect of government expenditure on economic growth by decomposing the former into recurrent and capital spending as well as total debt all as a ratio of GDP. It was found that the variables were co-integrated. In addition, it was observed that recurrent expenditure of the government and total debt exerted a significant and negative effect on economic growth in the long run [24]. assessed the extent of the impact of government spending on growth in Nigeria. Findings indicated evidence of co-integration between public spending and economic growth. Furthermore, it was observed that government expenditure particularly the recurrent component exerted a positive and significant influence on economic growth.

[1] appraised the relationship between public spending and economic growth in Nigeria. However, the study could not find evidence to support any significant relationship among the variables during the short run. In the long run, the study observed that all variables exerted a significant influence on economic growth. In a related study [40], assessed the degree of the impact of government expenditure on growth in Nigeria. Accordingly, findings indicated evidence of a significant positive impact of government spending on growth both in the long and short run. Still [13], modelled the government spending-growth nexus to test which theory between Wagner and Keynes holds in Romania [13]. attempted to x-ray the relationship that exists between government spending and economic growth using data from 1995 to 2018. Using the co-integration technique and granger causality, the study failed to establish evidence of a long-run relationship between the variables of interest. However, a bidirectional causal nature was observed by the study suggesting that public spending and growth cause each other. The study found strong evidence of bidirectional causality in the Romanian economy during the review period.

In China [18], appraised the impact of public healthcare expenditure on economic development. A spatial Durbin model using panel data from 31 provinces in China was tested with data covering 2005–2017. The study found that economic growth is significant and positively responsive to changes in healthcare expenditure but cautioned that whilst the direct effect exerted a significant impact on growth, the same cannot be said of the indirect effect [19]. utilized the OLS methodology on data spanning the period 1995–2018 to assess the extent to which government spending affected economic growth in Nigeria. Accordingly, the findings of the study indicated among other things that government spending had a significant negative impact on growth in Nigeria in the period of consideration. Employing quarterly data from the Indonesian economy for the period 2009Q3-2019Q3 [41], assess how government expenditure in micro, small and medium enterprises (MSMEs) has tended to promote economic growth. Accordingly, the findings of the study revealed that public spending is generally growth-enhancing but statistically insignificant in the specific case of MSMEs in Indonesia during the sample period.

[42] investigated the impact of government consumption spending in Saudi Arabia from 1985 to 2019. The granger causality technique and the ARDL model were used to investigate the contemporaneous relationship between the relevant variables. The study found evidence of a significant positive impact of government consumption spending on economic growth in the short run, but no relationship could be established in the long run. Similarly, bidirectional causality was discovered between government consumption spending and growth. As a result, the study recommended that the size of government spending be reduced to stimulate the growth of the private sector, thereby accelerating real GDP growth in the Kingdom of Saudi Arabia.

[43] conducted a similar study, examining the impact of sectoral public spending on social services such as health and education on economic growth over the period 1985–2018. Similarly, investment spending, imports, and exports were modelled using the ARDL technique for short and long-run analysis. As a result, the study discovered long-run relationships between the dependent and explanatory variables. In particular, the study discovered that government spending on education, investment, and exports had a significant positive impact on growth in the long run, whereas government spending on health and other components, as well as imports, had a significant negative impact. On the contrary, the study found no short-run relationships between these variables and growth. However, a unidirectional causality was discovered linking government spending on health and education to Saudi economic growth. As a result, the study advocated for prudence in the sectoral allocation of government spending to maximise the positive effect of government spending on growth in Saudi Arabia.

[44] employed the ARDL model to assess how public expenditure helps to foster inclusive growth through a reduction in poverty and inequality in developing countries covering the period 2000–2018. Findings indicated a significant positive impact of public spending on growth in developing countries [45]. assessed the extent to which controlling corruption will make public spending positively impact economic growth using data from 16 emerging economies of Asia during the period, 2002–2019. The GMM technique in the context of the threshold model was utilized by the study. The study found that controlling corruption in government expenditure exerted a significant negative effect on growth. However, the study noted that interacting government expenditure with control of corruption tends to reduce the degree of the negative impact. Furthermore, the study observed two threshold values of −0.61 and 0.01 for corruption control and concluded that public spending can stimulate growth in the emerging economies of Asia if the effort made to control corruption exceeds the minimum threshold value of 0.01.

## 3 Methodology

### 3.1 Theoretical framework

The model developed by neoclassical theorists led by Solow is adopted for the study. The model focuses on how the employment of factor inputs results in output productivity. Using the Cobb-Douglas production function the Solow model can be specified in equation (1) as thus:

(1) $$Y=AK^{\beta }L^{\alpha }$$

where: Y = total productivity, L = labour inputs, K refers to capital inputs while A encompasses total factor productivity. Also, α and β represent elasticities of output in labour and capital respectively. In adopting this model, the presence of A is a key factor influencing the economic level of output and for this reason, it is often referred to as TFP. From equation (2), L and K in the model were dropped and A takes the form

(2) Yt=f(δ)

Where δ is the vector of the explanatory variables expanded to accommodate all sectoral government expenditure variables.

### 3.2 Model and data

In this section, economic development is captured by HDI and specified as a function of sectoral government expenditure and a set of control variables

(3) $$HDI_{t}=f\left(GEX_{t},Z_{t}\right)$$

where: *HDI* = human development index, *GEX* stands for *GEXA, GEXE, GEXS*, and GEXT. Where *GEXA* = expenditure by government on administrative services, *GEXE* = expenditure by government on economic services, *GEXS* = expenditure by government on social and community services, *GEXT* = expenditure by government on transfer services. *Z* is a set of control variables including population change (*POP*), school enrolment (*SE*), inflation (*INF*) and oil price (*OP*). Estimating equation (3) in logarithmic stochastic term yields equation (4)

(4) $$HDI_{t}=\beta_{0}+\beta_{1}GEX_{t}+\beta_{2}Z_{t}+\mu_{t}$$

Where β_0_–β_2_ are constant and coefficients to be estimated respectively. Finally, μ refers to the error term. In what follows, the ARDL model is employed for the analysis. This model has been considered superior to other co-integration techniques in that it permits the inclusion of uneven lag order. It also allows different stationarity say I (0) and I (1). Also, the model is ideal for a small sample size with less than 50 observations. Additionally, the estimation incorporates in a single model the long-run and short-run simultaneously. The model was initially credited by Ref. [46] and later was expanded by Ref. [22] with a substantial contribution from Ref. [47]. The interest in this model for the current study was predicated on preliminary studies and also in line with several extant studies in the literature [2,25,40,[48], [49], [50]].

(5) $$\Delta HDI_{t}=\alpha_{0}+\sum_{i=1}^{K}\alpha 1i\Delta HDI_{t-1}+\sum_{i-1}^{K}\alpha 2i\Delta GEX_{t-1}+\sum_{i=1}^{K}\alpha 3i\Delta POP_{t-1}+\sum_{i=1}^{k}\alpha 4i\Delta SE_{t-1}+\sum_{t-1}^{k}\alpha 5i\Delta INF_{t-1}+\sum_{i=1}^{K}\alpha 16i\Delta InOP_{t-1}+\beta_{1}HDI_{t-1}+\beta_{2}GEX_{t-1}+\beta_{3}POP_{t-1}+\beta_{4}InSE_{t-1}+\beta_{5}INF_{t-1}+\beta_{6}OP_{t-1}+\varepsilon_{t}$$

Where *POP, SE, INF* and *OP* represent population change, school enrolment, inflation and oil price respectively. In developing the model [22], created lower and upper bounds used to test for the presence of co-integration between variables. As a result, a predetermined F-statistic is normally obtained through the Wald test from the ARDL procedure and this F-statistic is compared to the critical bounds. Accordingly, the null hypothesis cannot be rejected if the F-statistic falls below the lower bound and rejected if the value of the F-statistic exceeds the upper bound region. If the F-statistic lies between the lower and upper bounds, the test will be regarded as inconclusive [22,47]. Therefore, if the F-statistic suggests the presence of long-run equilibrium through cointegration relation, the short-run model is estimated using the error correction technique with predetermined variable lags.

(6) $$\Delta HDI_{t}=\varphi_{0}+\sum_{i=1}^{K}\varphi 1i\Delta HDI_{t-1}+\sum_{i-1}^{K}\varphi 2i\Delta GEX_{t-1}+\sum_{i=1}^{K}\varphi 3i\Delta POP_{t-1}+\sum_{i=1}^{k}\varphi 4i\Delta SE_{t-1}+\sum_{t-1}^{k}\varphi 5i\Delta INF_{t-1}+\sum_{t-1}^{k}\varphi 6i\Delta OP_{t-1}+\lambda ECT_{t}$$

where $\alpha_{0}$ and φ_0_ are the constant; $\alpha_{i}and\varphi_{i}$ are the parameters, Δ is the difference operator, K and p are the optimal lag lengths, t captures the time trend, ECT implies the error correction technique, and λ captures the speed of adjustment that regulates any disequilibrium between the short and the long-run. Results from Equation (6) are shown in Table 6

**Table 1:** Descriptive statistics of variables.

	HDI	GEXA	GEXE	GEXS	GEXT	INF	OP	POP	SE
Mean	−0.7115	5.7543	5.2960	5.0708	5.9916	2.4063	3.9070	0.9739	3.5484
Median	−0.7434	6.6554	6.1497	5.8096	6.4828	2.4366	4.0287	0.9809	3.5547
Maximum	−0.1743	7.6159	7.0264	7.1602	7.9772	3.9213	4.5571	1.0167	4.0290
Minimum	−0.7985	0.5364	0.3220	−0.0833	2.4849	1.6841	3.0398	0.9149	3.1592
Std. Dev.	0.1350	2.0993	2.0688	2.1936	1.6418	0.4899	0.5384	0.0303	0.2629
Skewness	2.9891	−1.3769	−1.4823	−1.3205	−1.0838	1.1418	−0.2901	−0.4923	0.0086
Kurtosis	12.5943	3.5097	3.6170	3.5058	3.0018	5.2500	1.6302	2.1443	1.7477

Source: Authors' computations, 2023.

**Table 2:** Multicollinearity test. Panel A: Correlation matrix. Panel B: VIF.

	HDI	GEXA	GEXE	GEXS	GEXT	INF	OP	POP	SE
HDI	1.0000								
GEXA	0.4301**	1.0000							
GEXE	0.4177**	0.9835***	1.0000						
GEXS	0.4351**	0.9885***	0.9715***	1.0000					
GEXT	0.4346**	0.9795***	0.9503***	0.9833***	1.0000				
INF	−0.0188	−0.0347	−0.0528	−0.0332	−0.0504	1.0000			
OP	0.5550***	0.8233***	0.7640***	0.8188***	0.8173***	−0.1157	1.0000		
POP	0.1033	0.2359	0.2370	0.2198	0.1508	0.0320	0.5211***	1.0000	
SE	0.4196**	0.7445***	0.6608***	0.7553***	0.7970***	−0.0277	0.8403***	0.2426	1.0000

Variables	Model 1	Model 2	Model 3	Model 4
	VIF 1/VIF	VIF 1/VIF	VIF 1/VIF	VIF 1/VIF
GEXA	4.5			
	0.2			
GEXE		2.8		
		0.3		
GEXS			4.4	
			0.2	
GEXT				5.0
				0.2
INF	1.5	1.5	1.4	1.4
	0.6	0.6	0.7	0.
OP	6.2	4.1	6.0	7.4
	0.1	0.2	0.2	0.1
POP	2.0	1.9	2.2	2.5
	0.5	0.5	0.5	0.4
SE	2.7	2.7	2.7	2,7
	0.3	0.3	0.4	0.4
Mean VIF	3.4	2.6	3.3	3.8

*, ** and *** stand for 10%, 5% and 1% level of significance respectively. Source: Authors' computations, 2023.

**Table 3:** Stationarity test.

	ADF			PP			KPSS		
Variables	Constant	Constant & trend	Remark	Constant	Constant & trend	Remark	Constant	Constant & trend	Remark
*HDI*	−4.49***	−5.79***	I (0)	−4.50***	−5.79***	I (0)	0.65	0.068**	I (0)
*GEXA*	−6.71***	−6.63***	I (1)	4.72***	−5.50***	I (1)	0.67	0.06	I (1)
*GEXE*	−6.02***	−6.22***	I (1)	−6.02***	−6.35***	I (1)	0.23	0.11***	I (1)
*GEXS*	−5.09***	−5.39***	I (1)	−5.12***	−10.05***	I (1)	0.65***	0.21***	I (1)
*GEXT*	−7.99***	−6.19***	I (2)	−4.07***	−6.51***	I (1)	0.65	0.19	I (0)
*INF*	−3.42***	−4.59***	I (0)	−2.90**	−3.37*	I (0)	0.42***	0.13***	I (0)
*OP*	−5.79***	−5.69***	I (1)	−5.82***	−5.69	I (1)	0.11	0.11	I (1)
*POP*	−3.11**	−4.39***	I (2)	−3.06**	−3.28*	I (1)	0.15***	0.15***	I (1)
SE	−9.97***	−6.36***	I (1)	−10.58***	−10.57***	I (1)	0.29***	0.12***	I (0)

*, ** as well as *** shows 10%, 5% as well as 1% level of significance respectively. Source: Authors' computations, 2023.

**Table 4:** Results of lag lengths from Equation (5). *Panel A, Model 1(F(*HDI*|*GEXA INF OP POP SE)*). Panel B, Model 2 (F(*HDI*|*GEXE INF OP POP SE)*). Panel C, Model 3 (F(*HDI*|*GEXS INF OP POP SE)*). Panel D, Model 4 (F(*HDI*|*GEXT INF OP POP SE)*)*.

Lag	LogL	LR	FPE	AIC	SIC	HQ
0	44.51827	NA	1.08e-10	−5.925888	−5.665142	−5.979483
1	127.3531	76.46289*	1.71e-13*	−13.13124	−11.30602	−13.50641
2	2138.284	0.000000	NA	−316.9668*	−313.5771*	−317.6635*

Lag	LogL	LR	FPE	AIC	SIC	HQ
0	−575.1240	NA	28,250,056	34.18377	34.45312	34.27563
1	−472.4031	163.1450*	578136.6	30.25900	32.14451*	30.90202*
2	−432.4180	49.39336	564017.4*	30.02459*	33.52624	31.21875

Lag	LogL	LR	FPE	AIC	SIC	HQ
0	−574.8747	NA	27,838,736	34.16910	34.43846	34.26096
1	−461.6862	179.7700	307785.8	29.62860	31.51410*	30.27161*
2	−418.8289	52.94132*	253592.6*	29.22523*	32.72688	30.41939

Lag	LogL	LR	FPE	AIC	SIC	HQ
0	−607.4717	NA	1.89e+08	36.08657	36.35593	36.17843
1	−495.5305	177.7890	2,253,517.	31.61944	33.50495*	32.26245
2	−443.8878	63.79398*	1,107,409.*	30.69928*	34.20093	31.89344*

* implies lag order selected by the criterion. Source: Authors' computations, 2023.

**Table 5:** Panel a. Bounds tests results. Panel B. Critical value bounds.

Model	*K*	F-statistic	Remarks
*(F(*HDI*|*GEXA INF OP POP SE))***	*5*	5.81	reject H_0_
*(F(*HDI*|*GEXE INF OP POP SE))***	*5*	5.78	reject H_0_
*(F(*HDI*|*GEXS INF OP POP SE))***	*5*	5.77	reject H_0_
*(F(*HDI*|*GEXT INF OP POP SE))***	*5*	5.76	reject H_0_

*K*	10%		5%		1%	
*5*	I (0)	I (1)	I (0)	I (1)	*I(0)*	*I(1)*
	2.26	3.35	2.62	3.79	3.41	4.68

**, * as well as *** connotes 5%, 10%, as well as 1% levels of significance respectively. Source: Authors' computations, 2023.

**Table 6:** Estimated models. Dependent variable: HDI.

Independent Variables	Model1	Model2	Model3	Model4
**Short-run**				
D (GEXA)	−0.000021 (−0.70)			
D (GEXE)		0.001428 (0.11)		
D (GEXS)			−0.000052 (−1.05)	
D (GEXT)				−0.00002 (−1.03)
D (INF)	0.00011 (0.16)	0.00033 (0.29)	0.000123 (0.19)	0.000123 (0.18)
D (OP)	0.00282*** (2.64)	0.00388*** (3.51)	0.00318*** (2.89)	0.00297*** (3.13)
D (POP)	−0.31233 (−1.41)	−0.41682* (−1.83)	−0.40906 (−1.64)	−0.39124 (−1.65)
D (SE)	−0.00056 (−0.67)	−0.00059 (−0.45)	−0.00054 (−0.65)	−0.000617 (−0.73)
ECM(-1)	−0.97164*** (−5.73)	−0.98869*** (−6.23)	−0.95449*** (−5.73)	−0.96671*** (−5.93)
**Long-run**				
GEXA	−0.00002 (0.66)			
GEXE		0.00144 (0.11)		
GEXS			−0.00005 (−0.97)	
GEXT				−0.00002 (−0.96)
INF	0.000113 (0.16)	0.000341 (0.30)	0.000135 (0.19)	0.000127 (0.18)
OP	0.002902** (2.26)	0.003925*** (3.27)	0.003327** (2.42)	0.003069*** (2.69)
POP	−0.321446 (−1.32)	−0.421595* (−1.86)	−0.428569 (−1.50)	−0.404711 (−1.53)
SE	−0.000586 (−0.66)	−0.000596 (−0.44)	−0.000566 (−0.64)	−0.000638 (−0.72)
C	1.215468** (2.00)	0.186258 (0.31)	1.481084** (2.09)	1.427300** (2.16)
Adj. R^2^	0.30	0.41	0.31	0.45
DW	2.0	1.99	2.0	2.1
Breusch-Godfrey	0.61^a^	0.43^a^	0.51^a^	0.59^a^
Breusch-Pagan-Godfrey	0.22^a^	0.43^a^	0.15^a^	0.17^a^
Ramsey RESET	0.14^a^	0.34^a^	0.5^a^	0.22^a^
ARCH	0.91^a^	0.91^a^	0.92^a^	0.93^a^

t-statistic in brackets and ***,**,* signifies 1%, 5% and 10% significance levels, in that order. Source: Author's Computation,2023.

a indicates Probability F-Statistic.

The study employed annual data on HDI and government sectoral expenditure namely social services, administration, transfer services and economic services. It also employed population change, school enrolment, inflation and oil price spanning the period 1986–2021. All four government expenditure components were culled from the various issues of the annual report and statement of account as well as the statistical bulletin of the Central Bank of Nigeria. Population change, school enrolment, inflation and oil price were sourced from the World Development Indicator of the World Bank while data on HDI were sourced from United Nations Development Programme (UNDP) database.

## 4 Discussion of empirical results

Before estimating the model, we examined the descriptive statistics of the variables in our study. The results presented in Table 1 show that for all variables, the standard deviation is low, which means the data are clustered around the mean and have fewer extreme values. This indicates that the sample mean is close to the true mean of the overall population. Additionally, the range of all variables' mean and median values is within the minimum and maximum values, indicating a high level of consistency among the variables. However, the kurtosis of all variables is slightly higher than normal, suggesting that they do not have a normal distribution. This observation is supported by the positive or negative skewness values of the variables, indicating that they are skewed in either a positive or negative direction. Nonetheless, we can conclude that the variables are platykurtic since their kurtosis values are less than 3.

We then use the correlation matrix to see if the variables are suffering from multicollinearity. Table 2 shows that all variables are not highly correlated except GEXA, GEXE, GEXS, and GEXT, which are all government spending and are used in separate models. With the exception of INF and POP, Table 2 shows that all other variables have a positive and significant correlation with HDI. Nonetheless, we found an insignificant negative correlation between government sectoral expenditure (GEXA, GEXE, GEXS, and GEXT) and inflation when each pair of independent variables is considered. We also find that government sectoral expenditure (GEXA, GEXE, GEXS and GEXT) and population change (POP) are not significantly correlated. However, there is a positive and significant correlation between government sectoral expenditure (GEXA, GEXE, GEXS and GEXT) and oil price (OP). A positive and significant correlation between government sectoral expenditure (GEXA, GEXE, GEXS and GEXT) and school enrolment (SE) was also found. Furthermore, no significant correlation was found between INF and OP, INF and POP, or INF and SE. Furthermore, there is a significant positive association between OP and POP, as well as between OP and SE, but no significant association between POP and SE was discovered. We used these findings to perform the Variance Inflation Factor (VIF) test to rule out multicollinearity among variables. Multicollinearity becomes an issue between variables with a VIF greater than 10.0, according to the rule of thumb. Panel B of Table 2 shows that the individual variable has a VIF of less than 10.0 in all models. As a result, the study concludes that none of the models exhibits multicollinearity.

It is crucial to conduct a unit root test on the variables to confirm their stationarity and determine the suitability of the methodology used in this study. The unit root test results, presented in Table 3, indicate that two variables are stationary at level I (0), while the others become stationary after the first difference I (1), as revealed by the ADF test. However, according to the Phillips-Perron (PP) test, two variables are stationary at level I (0), while seven variables become stationary after the first difference I (1). Additionally, the Kwiatkowski-Phillips-Schmidt-Shin (KPSS) test was performed, and all variables were stationary at level or integrated at order one I (1).

With stationarity confirmed, a vital condition for the ARDL technique has been satisfied. Table 4, therefore, presents lag lengths for the ARDL models to get around inaccurate results. Consequently, this study considers the minimum lag lengths from the AIC, SIC and HQ presented in Table 4.

After determining the appropriate lag lengths, we proceed to establish co-integration among the variables. The calculated F-statistic(s) are greater than the upper critical bound values at a 1% significant level, as shown in Table 5. When different types of government spending are interchanged in the ARDL model, we find co-integration relationships.

Surprisingly, the ARDL results from models 1–4 in Table 6 showed a statistically insignificant impact of government spending on human development both in the short and long run. Consequently, this indicates that the government spending-human development link may not exist in the case of Nigeria. In particular, the results demonstrated that expenditure by the government on administrative services, economic services, social and community services as well as transfer services has no connection with human development. These results contradict the findings of [32,34,[51], [52], [53]] which found significant effects of government spending (health, education, infrastructure, capital and recurrent) on human development. The economic implication of our result is that government spending on administrative, economic, social and community services will not contribute to human development in Nigeria. However, this development could be attributed to the country's high level of corruption, which diverts resources from social programs [45]. Concurs with this viewpoint. Additionally, although factors such as population change, school enrolment, and inflation do not account for human development in the country, the price of oil has a substantial impact on it both in the short and long term.

Nonetheless, the error correction terms in models 1–4 are correctly signed and highly significant at the 1% level, with coefficients ranging from 0.95 to 0.99. This level of relationship is to be expected if economic development and sectoral government spending are co-integrated. This high rate of adjustment implies that when disequilibrium between the short and long runs occurs, it takes approximately 95–99% of the time to correct it. Furthermore, the fact that ECMs are significant implies that government sectoral spending affected human development in the long run rather than the short run. This is because the ECMs are the residuals produced by the long-run dynamic estimates.

### 4.1 Diagnostic tests

Since the F-values (see Table 6) from the Breusch-Godfrey LM test in models 1–4 are not significant, the results in Table 6 show that there are no serial correlation issues in the estimated ARDL models. In addition, the Ramsey RESET test also shows that the models are properly specified as F-values are insignificant. Furthermore, none of the models exhibits a heteroskedasticity issue.

**Fig. 1:**
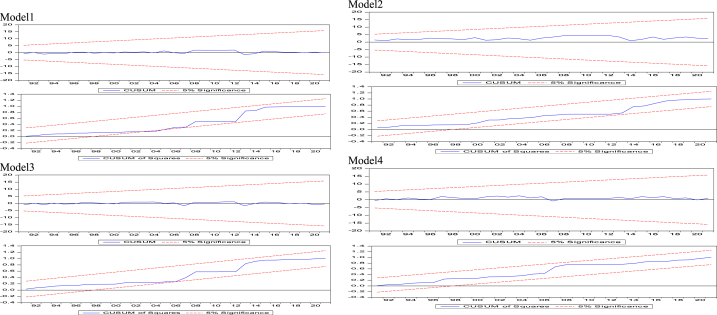
Model1. Model2. Model3. Model4.

Nevertheless, to be sure that the coefficients obtained are not biased, the cumulative sum (CUSUM) and cumulative sum of squares (CUSUMSQ) tests were examined. Obviously from Fig. 1, the regression coefficients are stable since the estimated coefficients do not exceed the base and top borders at the 5% significance level.

## 5 Conclusion and policy implications

This study examined the effects of government sectoral spending on human development in Nigeria. However, both in the short and long-run, results showed no link between government sectoral spending and human development. In this way, it is possible that the increase in government spending over the years has not yet been sufficient to support human development, or that the insignificant relationship is due to the country's high level of corruption, as corruption can divert resources away from social programmes. In terms of policy implications, the broad agenda for these empirical findings should be on how to increase government spending to facilitate human development.

Although the study discovered evidence of a long-run co-integrating equilibrium between the human development index and sectoral spending, further empirical investigation revealed that the effect of various sectoral government spending on the human development index is statistically insignificant both in the long and short run. The model's ability to present results in both short and long runs is important because theory suggests that the short run is more important in economic life because “in the long run, we are all dead” [54]. The results highlighted the importance of the three main components of the human development index, namely life expectancy at birth, adult literacy, and education enrolment, in ensuring a sustainable standard of living. These findings should alert policymakers in Nigeria to take action by implementing strategies like increasing public spending to address the decline in the human development index. Another option for Nigerian policymakers would be to combine targeted public spending in different sectors with direct investment, both domestic and foreign, to improve the current low level of the human development index in the country.

The study investigated the effects of government sectoral spending on human development. Although, the study found no connection between government sectoral spending and human development but this may be blamed on the high level of corruption in the county. Future studies can build on this study by investigating the effect of corruption in the link between government spending and human development since theoretical literature affirmed that government spending can promote economic development.

The study used annual time series data, which may have had a weaker impact than quarterly data due to the increased number of observations. Future studies may attempt to use quarterly data in this regard. Furthermore, the ARDL model used does not account for any shocks in human development caused by changes in government spending. As a result, future studies may employ models such as VAR and ARCH.

## Author contribution statement

Ademola Andrew ONABOTE: Conceived and designed the experiments; analyzed and interpreted the data; contributed reagents, materials, analysis tools or data; wrote the paper.

Bright Onoriode OHWOFASA: Conceived and designed the experiments; analyzed and interpreted the data; wrote the paper.

Rotimi Ayoade OGUNJUMO: Analyzed and interpreted the data; contributed reagents, materials, analysis tools or data; wrote the paper.

## Additional information

No additional information is available for this paper.

## Declaration of competing interest

The authors declare that they have no known competing financial interests or personal relationships that could have appeared to influence the work reported in this paper.

## Figures and Tables

**Table A:** Measurement and Sources of Data.

Variables	Acronym	Measurement	Source(s)
Human development index	HDI	Involves educational attainment, life expectancy and per capita income.	UNDP, 2021
Government spending on administrative services	GEXA	Government expenditure on administrative services various years	CBN report
Government spending on economic services	GEXE	Government expenditure on economic services	CBN report on various years
Government spending on social and community services	GEXS	Government expenditure on social and community services	CBN report various years
Government spending on transfer services	GEXT	Government expenditure on transfer services	CBN report various years
Population change	POP	Population growth	WDI, 2023
School enrolment	SE	Secondary enrolment % of gross	WDI, 2023
Inflation	INF	Consumer prices annual %	WDI, 2023
Oil price	OP	Real average price ($/bbl)	World Bank, 2023
